# Binary dataset for machine learning applications to tropical cyclone formation prediction

**DOI:** 10.1038/s41597-024-03281-5

**Published:** 2024-05-03

**Authors:** Chanh Kieu, Quan Nguyen

**Affiliations:** Department of Earth and Atmospheric Sciences, Indiana University, Bloomington, IN 47405 USA

**Keywords:** Atmospheric dynamics, Scientific data

## Abstract

Applications of machine learning (ML) in atmospheric science have been rapidly growing. To facilitate the development of ML models for tropical cyclone (TC) research, this binary dataset contains a specific customization of the National Center for Environmental Prediction (NCEP)/final analysis (FNL) data, in which key environmental conditions relevant to TC formation are extracted for a range of lead times (0–72 hours) during 1999–2023. The dataset is designed as multi-channel images centered on TC formation locations, with a positive and negative directory structure that can be readily read from any ML applications or common data interface. With its standard structure, this dataset provides users with a unique opportunity to conduct ML application research on TC formation as well as related predictability at different forecast lead times.

## Background & Summary

Predicting the formation of a tropical cyclone (TC) is an important task at operational weather centers. Early prediction of tropical cyclogenesis (TCG) can help forecasters issue proper warnings and prepare for various risk management. For practical purposes, TCG prediction is currently treated as a classification (categorical) problem, with an expected outcome as a yes or no for a TCG event at a certain lead time. In the current operational setting, this yes/no TCG forecast is often associated with a probability at 1, 2, or 3-day lead time as routinely provided by, e.g., the National Hurricane Center (NHC) during TC main seasons. From the machine learning (ML) perspective, TCG prediction therefore belongs to a class of logistic regression problems that have been well-developed in ML applications.

Despite this well-defined formulation of the TCG problem, predicting TCG has been always challenging to date. This difficulty in TCG prediction is due to complex multi-scale interactions among different dynamical and thermodynamic processes in the atmosphere during the early formation period. In general, these processes are highly nonlinear and vary across ocean basins that no single dominant mechanism could operate in all oceans. In fact, verification of real-time TCG forecasts from different physical-based models^1–3^ showed that current global models could achieve a probability of detection in the range of 0.3–0.5 at 2–3 day forecast lead time, but the false alarm rate is also unexpectedly high in the range of 0.5–0.7. There has been some steady progress in the success ratio for TCG forecast, but this progress has been slow across ocean basins, models, and forecast lead times^3,4^.

The rapid development of advanced ML algorithms opens up a new opportunity for forecasters and researchers to explore new methods in TCG prediction^5^. Unlike the traditional approach based on vortex-tracking algorithms from the model output, ML algorithms can allow one to search and learn different features from the environment that have the most impacts on the TCG processes in the same way as an experienced forecaster could learn from a weather map. By training ML models on a large number of input data, ML models can recognize or look for the most important environmental features related to TCG at different forecast lead times or ocean basins.

A key factor determining the successful development of an ML model for TCG prediction is rooted in the quality of training data. Generally, a good dataset is the very first priority that one needs for an ML model to achieve an expected accuracy. In ML development, a dataset is considered to be of high quality if it meets several basic requirements including (i) comprehensiveness, (ii) relevancy, (ii) consistency, and (iv) uniformity^6–8^. Among these requirements, comprehensiveness is the most difficult for the TCG prediction problem. While the current climate data is enormous, the data that can be actually used for TCG models is limited due to the rarity of TCs and a short period of high-quality climate datasets during the post-satellite era^9^. Assuming there are O(10^2^) TCs annually at the global scale, a 20-year period can provide at most  O(10^3^) TCG events. This number will be cut down further if one focuses on a specific subdomain, and so the dataset for TCG is considered to be small by all means of current ML training standards.

In addition to the quality of the climate dataset, it is of equal importance to have a good set of environmental variables that can encode signals of TCG. The process of selecting the best input variables, often known as feature engineering in ML, plays a key role in the development of ML models that do not have sufficiently large training data. This feature engineering is especially useful for TCG prediction due to the lack of high-quality climate data and the limited number of TCG events every year as mentioned above. Previous observational and modeling studies have captured a number of key environmental conditions for TCG such as warm sea surface temperature, low vertical shear, moist lower troposphere^10–16^. While these variables have been shown to be important from numerous modeling and observational studies, they turn out to be insufficient to guarantee TCG in real-time forecast. In particular, their relative importance changes from one ocean to the other, making it hard to generalize for operational TCG prediction at present.

Because a good set of training data and input features are the backbone for the success of ML model development, it is essential to generate a dataset for TCG research that helps ML models better comprehend data and memorize past information for TCG prediction. In this study, we present a binary dataset customized specifically for TCG prediction, which can be readily used in any current ML algorithm or data loader interface. Recall that the common binary classification is a supervised problem, which scans through all training data to search for dominant features that can be used to predict possible classes. For TCG prediction, our classification problem is therefore designed as a yes/no TCG event at a given forecast lead time, along with an associated probability for the yes/no prediction. From this perspective, it is required to create a binary dataset that could contain yes/no TCG events for a range of lead times such that ML models can be trained on. Such a dataset will not only help understand how effectively an ML model can detect TCG events from a given input, but it is also of great use for other research such as understanding TCG predictability relative to physical-based models, or quantifying the relative roles of different environmental factors in TCG processes.

## Methods

### Input data

To generate a binary dataset for TCG prediction, the National Center for Environmental Prediction (NCEP) final analysis (FNL^17^,) data at a resolution of 1 × 1 degree during the 1999–2023 period was used. For this dataset, our main areas of focus are the three major ocean basins where TC activities are most prominent including the northwestern Pacific, northeastern Pacific, and North Atlantic Ocean during the main TC season from May to November. Within each region, nine key meteorological variables are extracted from the gridded FNL data, which are most relevant to the TCG processes. These variables include all three wind components, absolute vorticity, relative humidity, temperature, geopotential height, surface temperature, convective available potential energy (CAPE), and tropopause properties (see Table 1). While these variables are chosen based on their potential impacts on TCG as shown in the previous studies^18–23^, how effective they are or their relative importance among those variables in detecting TCG by ML models at different forecast lead times is still elusive. Likewise, these variables are provided at all 19 NCEP/FNL pressure levels, yet it is not currently known which level would provide the maximum information about TCG. Thus, all pressure levels for these variables are included in our binary dataset, which can serve as input channels for any ML model development.

**Table 1:** A list of variables extracted from the NCEP/FNL data for the TCG binary dataset.

Variable	Vertical levels	Remarks
Absolute Vorticity (s^−1^)	19	native NCEP pressure levels
Relative Humidity (%)	19	native NCEP pressure levels
Temperature (K)	19	native NCEP pressure levels
Geopotential Height (m)	19	native NCEP pressure levels
Vertical Wind (Pa s^−1^)	19	native NCEP pressure levels
Zonal wind (m s^−1^)	19	native NCEP pressure levels
Meridional wind (m s^−1^)	19	native NCEP pressure levels
CAPE surface (J kg^−1^)	1	surface level
Surface Temperature (K)	1	surface level
Tropopause Temperature (K)	1	tropopause level
Tropopause height (m)	1	tropopause level

While using a single NCEP/FNL reanalysis data is certainly a limiting factor for our binary dataset, we note that the NCEP/FNL reanalysis is useful in two important aspects. First, it can serve as input data for a pre-trained model that can be further refined later on for different datasets or downstream applications. This process, known as transfer learning, can help speed up later training with any other datasets such as the Modern-Era Retrospective Analysis for Research and Applications, Version 2 (MERRA-2) or European Reanalysis (ERA-5).

Second, the 2008–2023 period contains GRIB2 data format produced by the same NCEP Global Data Assimilation System (GDAS), which synthesizes observational data from various sources and analyses. As such, this dataset provides a consistent multivariate, spatially coherent state of the global atmosphere at a homogeneous resolution, ensuring the consistency and the uniformity of the training dataset for ML models. Moreover, this dataset contains some diagnostic variables such as Convective Available Potential Energy (CAPE), surface fluxes, or some tropopause properties such as tropopause height or temperature that are directly relevant to TCG. These advantages of the NCEP/FNL dataset allow ML models to detect proper TCG features during both training and validation steps.

Although the NCEP/FNL data includes new types of observation as well as data assimilation upgrades every year, we note that these annual changes in FNL data should have a minimum impact on the overall usability of our dataset for ML applications. This is because the main aim of our binary dataset in this study is to facilitate ML model development as well as future validation/comparison among different ML models, at least during the proof-of-concept model development. Of course, better reanalysis data would provide better training data and help ML to converge faster. Thus, our dataset should be considered only as preliminary learning that needs to be further fine-tuned via the so-called “transfer learning” as mentioned above. From this perspective, the need for a binary TCG dataset is more pressing for current ML applications in TC research than the annual change in the NCEP/FNL reanalysis data.

It should be noted that while a higher resolution NCEP/FNL data (0.25° × 0.25°) is available, this data is limited to the 2015–2023 period, while the 1 degree data has a longer period from 1999 to 2023. Because predicting TCG using the ML approach requires searching for a range of environmental features instead of using physical processes, it is important to have as long as possible a training dataset that could help identify those environmental features. Thus, we chose the NCEP/FNL at 1-degree solution for our binary dataset to increase the sampling and the ability of feature detection for ML applications, instead of a shorter dataset at a higher resolution.

For labeling TCG events, the International Best Track Archive for Climate Stewardship (IBTrACS^24,25^) was used for all TCG timing and locations. This is a well-calibrated data record of TC activities in global ocean basins and includes all information related to TCs during their lifecycle such as intensity, ocean basin, longitudes, latitudes, name, the stage of development, as well as their corresponding date and time at an interval of every 6 hours. Although there exist several other regional TC databases, we used the IBTrACS for our binary dataset so that TCG information in our dataset can be compared and/or validated among previous studies that used this IBTrACS database. Because IBTrACS incorporates TC data from many different agencies, we specifically used the US National Hurricane Center data for the Eastern Pacific and North Atlantic basins, and the Joint Typhoon Warning Center for the Northwestern Pacific basin. Given that TCG takes place over a large area, the uncertainty in the initial location of a TCG event among different best-track databases (of order O(10) km) should have minimal impact on quantifying environmental conditions for TCG, so long that the data domain size is large enough to include the entire TCG area.

### Algorithms

For our TCG dataset, we define a TCG event as the first time a storm was recorded as a Tropical Depression, with all subtropical cyclones excluded^5^. With this definition, the IBTrACS data is searched through and the first recorded location of each storm is used to create a set of positive-labeled TCG data. To further facilitate our data generation, all information relevant to a TCG event such as its longitudes, latitudes, date, and time are extracted and archived in a separate CSV database, which can be shared with different data interfaces for future upgrades or applications.

Given the global domain of the NCEP/FNL data and the CSV database containing all TCG information, we generated a positive-labeled dataset for TCG events by zooming into a fixed domain centering on each TCG event. Specifically, to create the TCG binary dataset, we pre-selected a domain of size 18 × 18 degrees around the TCG location and then extracted all variables in the NCEP/FNL data relevant to TCG in this domain. By design, all domains centered on the TCG events recorded in IBTrACS will be labeled as a positive part of our TCG binary dataset for three main ocean basins including North Atlantic (NA), Eastern Pacific (EP), and Northwestern Pacific (WP).

Regarding the negative-labeled data (i.e., the data with no TCG event), there are several different ways to produce it, depending on the objectives and the nature of the problem. For our TCG prediction dataset, we adopt a method that creates negative data for each corresponding positive TCG data point by choosing a region of the same size as the positive-labeled data but at a distance of 10 degrees apart (measured between two domain centers). Our choice of this negative domain is based on the physical nature of the TCG problem, which often focuses on why a TCG event occurs at one particular location but not other locations nearby at the same time. By designing the negative-labeled data this way, we remove the inhomogeneity in the temporal dimension (such as seasonal or weekly differences of the large-scale environment) between the negative and positive data pair, which helps focus on the environmental factors that govern TCG at the same time but in different locations.

Another way to produce negative-labeled data is to randomly choose a domain location during the dates/times that have no TCG. This negative-labeled data will lead, however, to two issues: (1) the number of negative-labeled data points will be much higher than that of the positive-labeled data points (because most of the days have no TCG), and (2) the negative and positive-labeled data now contain different temporal and spatial environments (for days with no TCG events, there is no good way to choose a negative-labeled domain, unless one fixes a specific geographical location). These issues make it harder to interpret or extract TCG processes. As such, this latter approach is not adopted for our TCG binary dataset herein, albeit it is useful for predicting TCG in practice.

Because the positive-labeled data based on TCG locations in the IBTrACS dataset could contain grid points that are too close to land, which prevent one from choosing an arbitrarily nearby negative-labeled data, we designed our algorithm to select negative-labeled data based on several criteria as follows:

1. The center of the negative-labeled data corresponding to a given TCG location is randomly chosen among the four quadrants around the center of the positive-labeled data;
2. The distance between the centers of the positive and negative-labeled data pair must be at least 10 degrees apart
3. No grid point on the negative-labeled domains overlaps land surface;
4. The northern edge of the negative-labeled domain size must not cross 30°N; and
5. The negative-labeled domains will not overlap each other in case there are multiple TCG events on the same date/time.

These conditions are based on the fact that the TC main formation area in all ocean basins should be over open ocean and generally between 5–30° degrees. Note also that for the date and time with multiple TCG events, the algorithm will generate each positive-labeled domain for each TCG event independently such that they do not overlap each other. Doing this way will ensure that each positive TCG data will always have its own negative data pair, thus maintaining the data balance for ML training as expected.

During the development of ML models for TCG prediction, we noticed one additional issue that is unique to TCG prediction. That is, occasionally some false positive TCG predictions (i.e., negative cases with no observed TCG events but were incorrectly predicted as positive by ML models) contain pre-existing TCs nearby. These nearby TCs could introduce some TC characteristics into the surrounding environments and confuse ML models in predicting TCG probability. As such, ML models may not tell if the environments favorable for TC development are from a pre-existing TC nearby or if these environmental conditions are applied to a new TC formation. To address this issue, we employed a modified vortex removal scheme for any case where a nearby TC is too close to a new TCG event that the TC-related information from the nearby TC could enter the domain of the new TCG event^26^. This vortex removal algorithm follows the same Kurihara 1993’s approach^27^, and has proven to be effective for eliminating TC vortices as shown in previous studies.

In practice, the co-existence of more than one TC at the same time and sufficiently close to a new TCG event, which requires our special treatment of vortex removal as described above, is very rare (<1% of all TC cases that we have in our dataset). In particular, we can always minimize the vortex removal procedures to avoid changing large-scale environments by simply choosing a different negative–labeled domain in different parts of each basin as outlined in Step 1 above. As such, the actual number of cases that we had to apply the vortex removal algorithm for our negative-labeled data is limited to only a few TCs (out of 1425 total cases), which include TC Barijat (2018), Wukong (2000), Mujigae (2009), Kompatsu (2016), and Mirinae (2017).

**Fig. 1:**
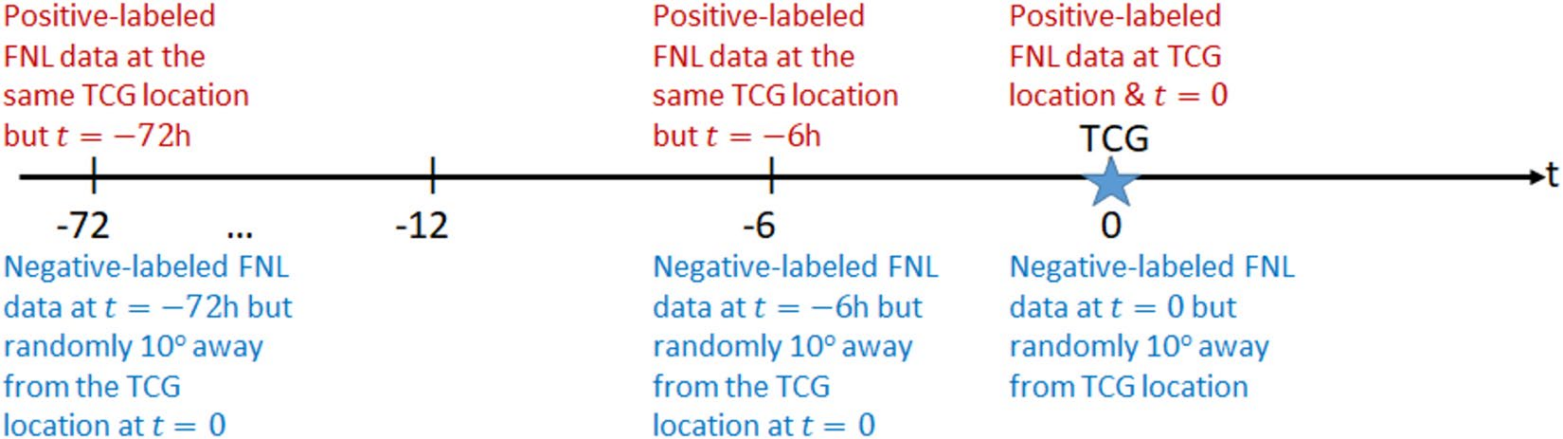
Illustration of the binary dataset generation for a TCG event at different lead times *τ* = −72, …, −12, −6, 0 h relative to the TCG moment (star).

With the goal of providing a dataset for not only detecting TCG events but also for predicting TCG at different lead times, the whole process of creating positive/negative pairs is repeated at an interval of 6 hours up to 72 hours *prior to* the TCG moment for the entire TCG records. Specifically, the procedures of producing a *τ*-lag time TCG binary data are identical to what described above, except that one has to look for the NCEP/FNL dataset that corresponds to a lagged time at *τ* hours relative to the actual genesis record (see Fig. 1), where *τ* = −72, …, −12, −6, 0 is the time (hours) relative to the TCG moment. Note that while the domain center for the positive-labeled data is always fixed at the same as the location of the TCG event for all lagged-time *τ*, the domain center for negative-labeled data is not. This is because, by design, the center of the negative-labeled domain is chosen randomly at 4 different quadrants around the domain center of positive-labeled data (conditioned on the 5 criteria listed above). Thus, positive-labeled data is continuously back in time, while negative-labeled data does not. This type of time-lagged data is particularly useful for studying TCG predictability in which one starts with a single time slice of input data and makes prediction *τ* hours in advance, without using any physical equation or past memory. Such a process is very similar to a numerical weather prediction model that produces a forecast based only on a single initial condition. As presented in our recent study^5^, the forecast skill of any ML model using this time-lagged data can help answer the question of how much predictability one can achieve for TCG prediction if one uses a pure ML model without any governing equations. Likewise, the time-lagged data can be also used for the development of recurrent neural network (RNN) architectures to improve further TCG prediction. By choosing different time slices as input for an RNN model, one can take into account some additional memory of the temporal evolution of the atmospheric state that can be useful for TCG prediction. Our time-lagged dataset serves this exact purpose for TCG prediction.

Note that for *τ* = 0, the TCG prediction problem will be reduced to TCG detection, which will be useful in practice from a different perspective. For example, one can train an ML model to detect a TCG event from model output when a global analysis or forecast data at any lead time is available. Similarly, one can also use the models to detect TCG from climate output, which can help project future TC activities beyond the traditional vortex-tracking methods. From this perspective, our binary TCG dataset can help address research questions related to either detecting TCG from a global analysis or predicting TCG at different forecast lead times.

Although the time-lagged dataset is designed for examining the TCG prediction skill at different forecast lead times, we should mention here that this dataset is also useful for examining the uncertainties in the timing of TCG recorded in IBTrACS. Generally, TCG timing can be always determined to the nearest six hours in IBTrACS. However, any such TCG timing contains some uncertainty to within 6–12 hours around the TCG moment due to different TC records by different operational agencies. Given such uncertainties in TCG timing, our time-lagged dataset back to 72 hours prior to the first recorded genesis time will therefore provide some additional information for sensitive analyses and examining the TCG prediction skill for any ML model or diagnostics.

## Data Records

**Fig. 2:**
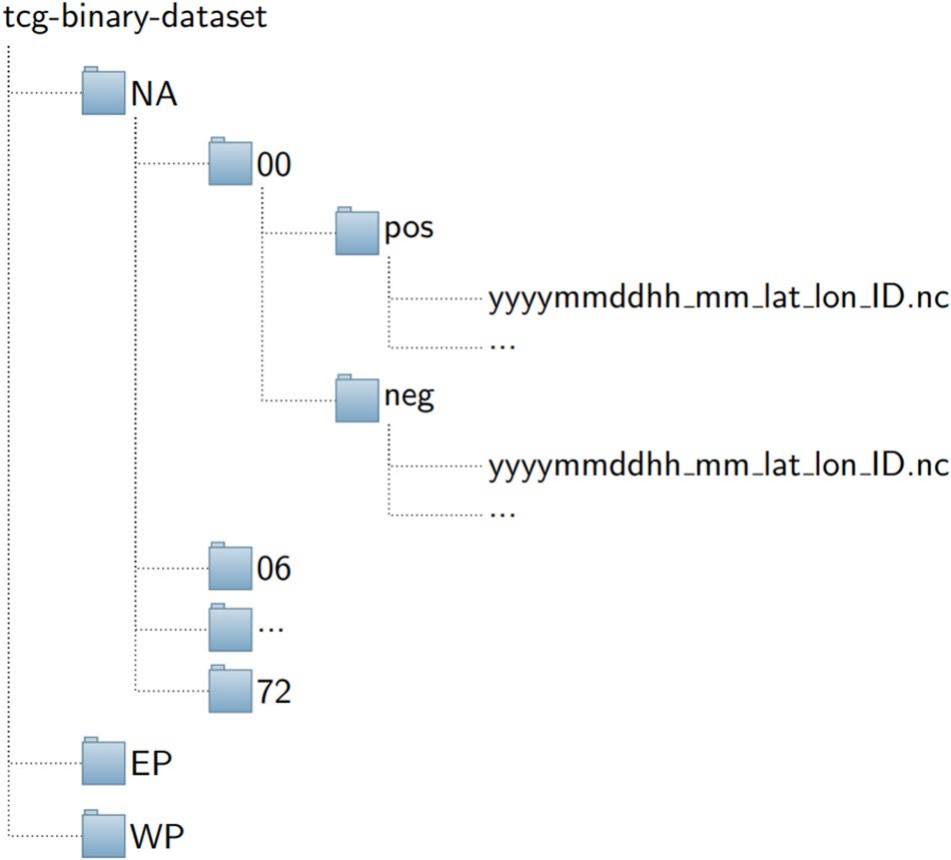
Directory structure of the binary TC formation dataset. Note that the parameters (lat,lon) in each file name indicate the latitude and longitude of the domain center where the data is centered. For the positive-labeled data, this (lat,lon) is the location of TC genesis valid at t = 0, which is the first time a genesis event is recorded in the best track data. For the negative-labeled data, this is the location where the data is extracted and valid at the same time as its corresponding positive-labeled data. For all other directories 12, 24,…,72, these are the time prior to the genesis moment.

To facilitate ML model development and training, we organize our TCG binary dataset according to three different ocean basins including NA, EP, and WP as the top-level directory structure as shown in Fig. 2. For each basin, the data is then stored in each TCG lagged time ranging from 0 to 72 hours, with each lag-time directory containing two sub-directories: one for positive-labeled data in a directory named “pos”, and the other corresponding negative-labeled data stored in a directory named “neg”. This data structure is common in many ML binary datasets, which are organized in such a way that facilitates common data interface loaders or ML frameworks such as Keras or Scikit-learn. For each positive/negative-labeled data file, all variables listed in Table 1 are extracted on all vertical pressure levels as in the original NCEP/FNL dataset, but they are reformatted in the NETCDF format centering on TCG locations. To ease the validation of the TCG binary dataset, each filename in pos/neg directory contains a string as yyyymmddhh_lat_lon_ID, where lat,lon denote the latitude and longitude of a TCG event, yyyymmddhh denotes the time of the TCG event, and ID is the 13-digit identification number corresponding to the TCG event in the IBTrACS record (i.e., the first column in the IBTrACS CSV-format data file). By naming all pos/neg data files this way, users can quickly track any TCG event by simply looking at the filename in each directory. Note that because the data is stored in the NETCDF format, users need to read the data using a standard package such as netCDF4 or xarray.

In the current version, our TCG binary dataset contains a total of 390, 448, and 587 TCG events during the 1999–2022 period in the NA, EP, and WP basins, respectively. This is a quite small set for training any ML models for each basin individually. However, one can always combine all basins into a single dataset to increase the sample size for ML model development. The scripts provided in our GitHub along with this dataset can be used to extend for any period or other reanalysis datasets of interest to users. In our future release of an upgraded TCG binary dataset, we plan to include a more complete dataset at higher resolutions. Full access to our TCG binary dataset is currently available on our Figshare data repository^28^.

## Technical Validation

**Fig. 3:**
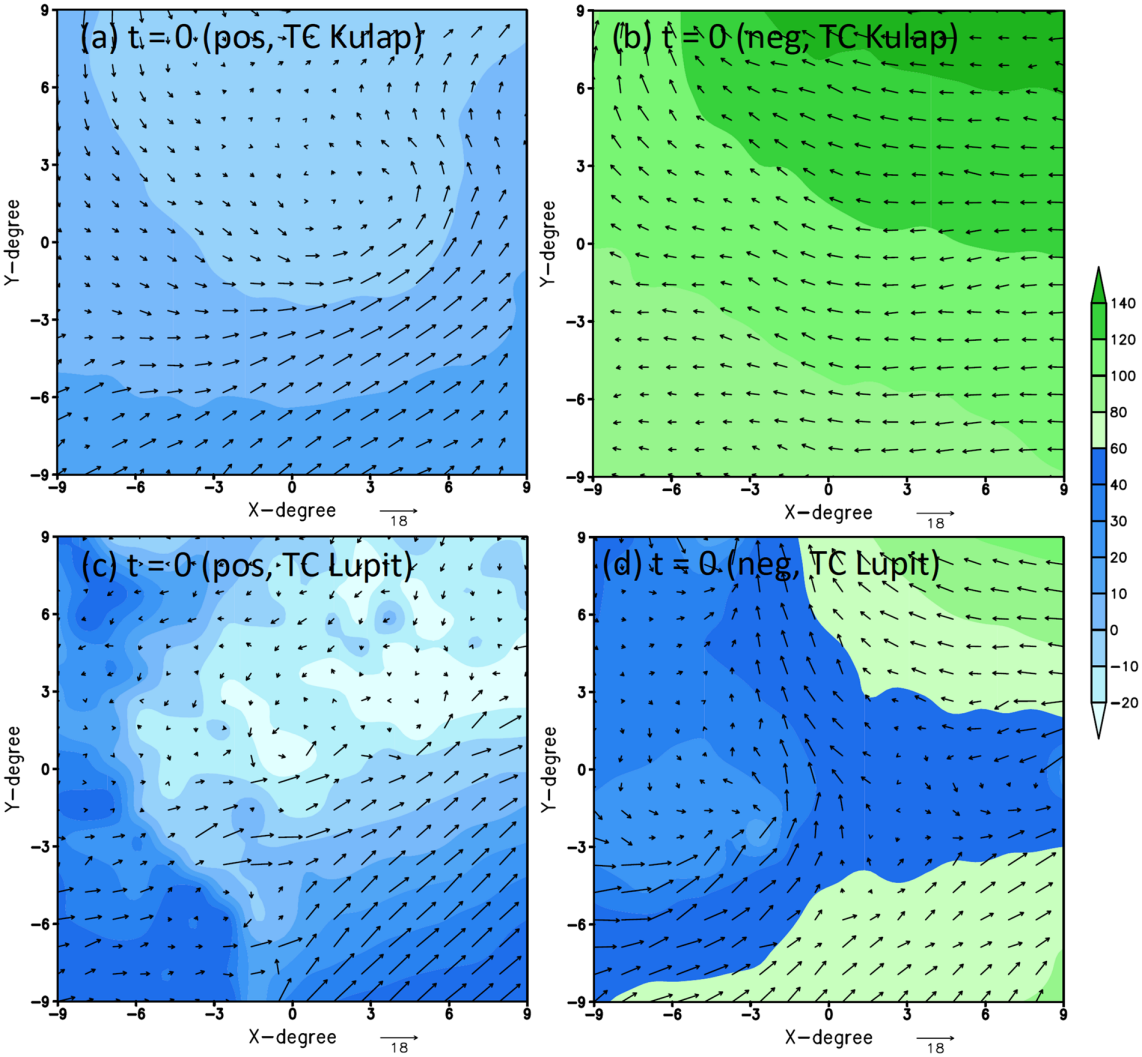
The horizontal cross sections at *z* = 950 hPa of the geopotential height perturbation (shaded, unit gpm) and the corresponding horizontal wind (vectors, unit ms^−1^) for the genesis of Typhoon Kupat valid at 1800 UTC, September 5, 2011 for (**a**) a positive sample, and (**b**) a negative sample valid at the same time; (**c,d**) similar to (**a,b**) but for Typhoon Lupit valid at 0600 UTC, August 02 2021.

Because we do not modify or introduce any new variable/coordinate transformation to the NCEP/FNL original data, all data quality and characteristics are fully preserved. The only technical validation that we have to carry out is to ensure that the positive/negative-labeled data are well paired with each other as designed. For this purpose, we plot several random samples of data pairs for the TCG events associated with Typhoon Kupat (2011) and Typhoon Lupit (2021) at different TCG lag times. Figure 3 shows an example of a positive and a negative-labeled data for these two typhoons valid at lag time *τ* = 0 (i.e., right at the TCG moment). As seen in Fig. 3, the positive-labeled data does capture a weak signal of these TCG events at the genesis moment for both samples (shown as the low geopotential perturbation at *z* = 950*hPa* and the corresponding cyclonic flow field). These genesis signals are most apparent for the near-surface field and quickly fade away at higher levels. This is expected because the tropical depression stage often displays only marginally weak TC signals from satellite and/or reanalysis datasets, with strong uncertainties in both location and strength. Compared to the negative data valid at the same times but located in different locations (Fig. 3b,d), one notices that the negative-labeled data do not display a clear signal of low pressure or geopotential perturbation as expected. Likewise, the flow field is also less organized, despite being extracted at the same time and close by. By construction, this negative-label data can therefore provide a reference for large-scale environments that do not support TCG, thus allowing ML models to learn different environmental features between positive and negative samples as designed.

**Fig. 4:**
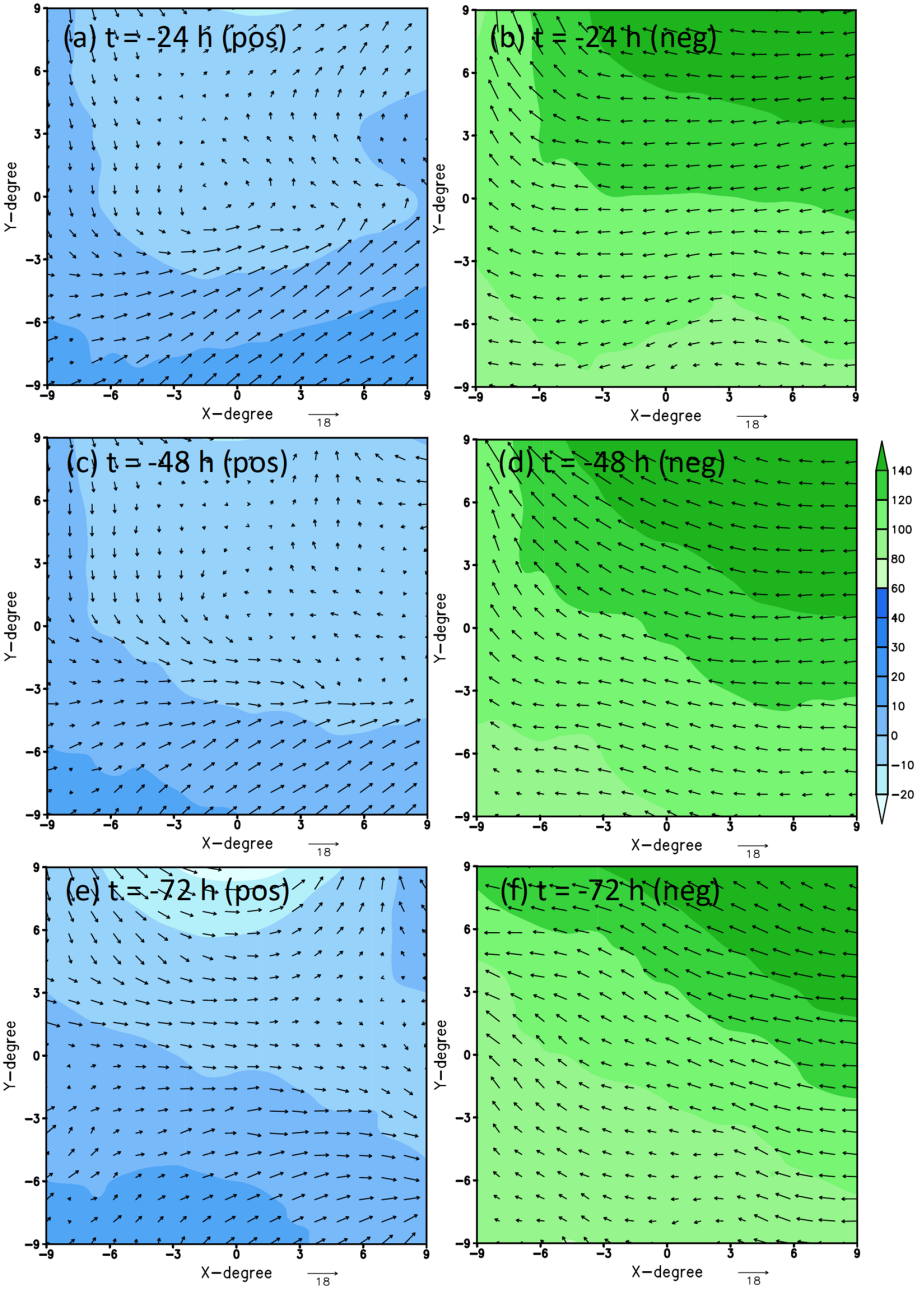
Similar to Fig. 2a,b but for 24, 48, and 72 h prior to the genesis of Typhoon Kupat.

**Fig. 5:**
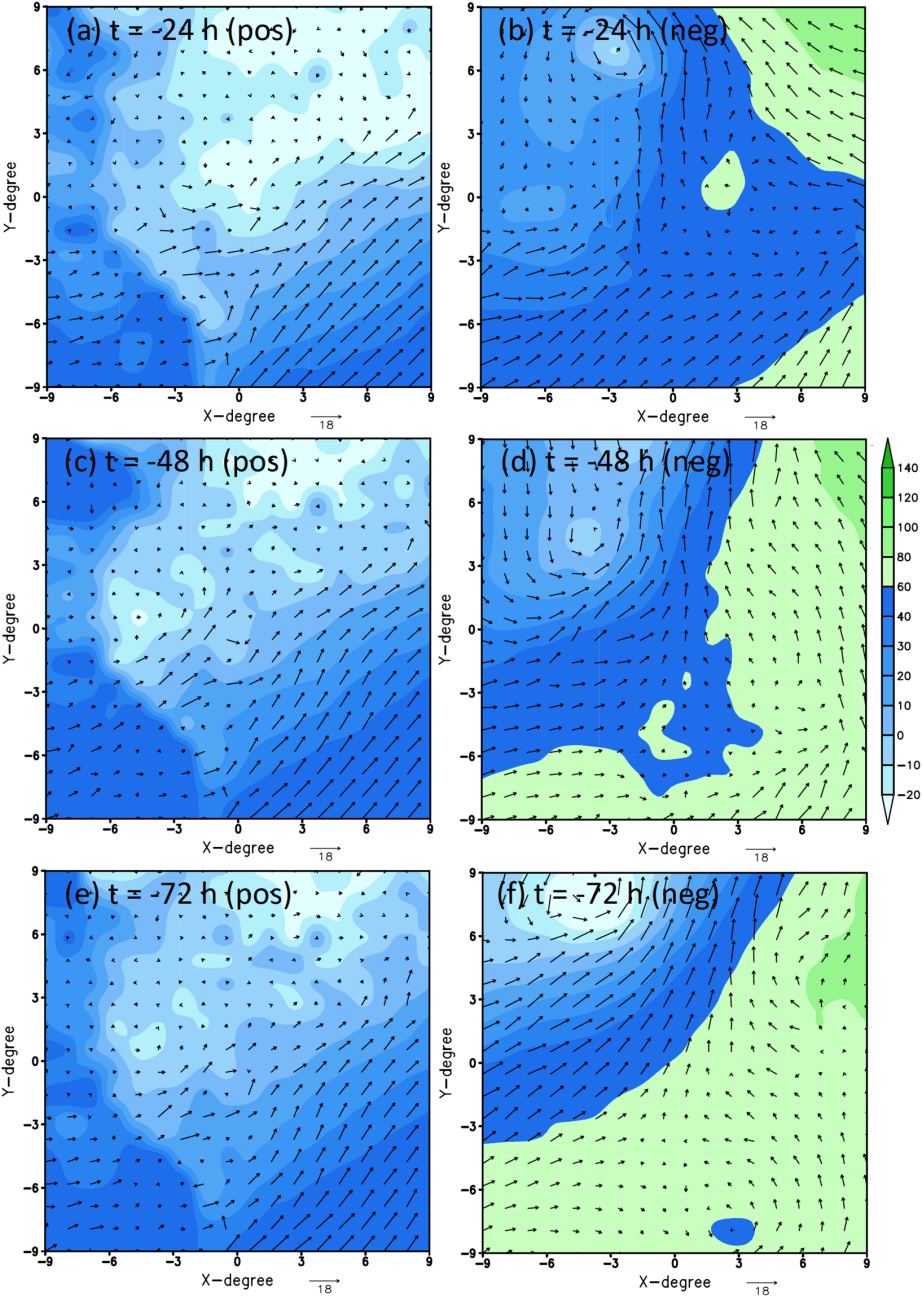
Similar to Fig. 2c,d but for 24, 48, and 72 h prior to the genesis of Typhoon Lupit.

Looking further back at 1, 2, and 3 days prior to the genesis moment at the same TCG location, one could also confirm that TCG signals become gradually less clear, even for the positive-labeled data. In fact, for a 2-day lag time, there is virtually no TCG signal for Typhoon Kupat (Fig. 4) while the TCG signal is very weak for Typhoon Lupit (Fig. 5). This deterioration of TCG signals with different lead times justifies the difficulty of predicting TCG at long lead times purely from memory as presented in our recent study^5^. Note that this behavior of less TC signal with a longer lag time is very typical for all TCG cases, which we could visually confirm at least for all cases that are randomly examined beyond the examples shown in Figs. 3–5. As such, the TCG binary dataset is well-designed for TCG research. Unlike the positive-labeled data, it should be noted again that negative-labeled data are not centered on a TCG location but randomly sampled at a fixed distance from its corresponding positive-labeled data. As such, the negative-labeled data do not always vary smoothly from one lag time to the next.

## Usage Notes

All of the positive/negative-labeled outputs provided in this TCG binary dataset are produced by the workflow and libraries included in our GitHub repository here https://github.com/kieucq/tcg-binary-dataset. This is a public repository with an open-source license, and so users can freely download, reproduce, or expand our TCG binary dataset. For the sake of completeness, we provide below some specific instructions to reproduce our dataset with the workflow provided in this repository. The key steps include:

Step 1. Clone our GitHub repository from https://github.com/kieucq/tcg-binary-dataset and save it to your working directory (hereinafter assuming that it is saved under dir_work).

Step 2. Download and create a directory that saves all NCEP/FNL reanalyses, which is assumed to be stored in an environment path variable dir_ncep. Note that under this location dir_ncep, each year must be saved separately under the same format for the directory name as yyyy, even if users have only one year;

Step 3. Download and save the best track dataset that is assumed to be stored by an environment path variable dir_tc. Note that the current support for our binary dataset is built on the IBTrACS dataset in the CSV format. Users who want to use a different tropical cyclone database can reformat their database such that TC center latitude, longitude, and date/time columns can be properly read in our script, using package pandas.

Step 4. Set up the domain size (domain_size), forecast lead time (lead_time), ocean basin (basin), distance between the center of the positive-labeled and negative-labeled domain center (dist), and output location where you want to save the output (dir_out). Note that the current basin support includes only three basins NA, EP, and WP.

Step 5. Run the script create_ncep_binary_stormcenter_grib1.py to process GRIB1 data using the following syntax:


python./create_ncep_binary_stormcenter_grib1.py --best-track $dir_tc --ncep-fnl $dir_ncep --basin $basin --leadtime $lead_time --domain-size $domain_size --distance $dist --output $dir_out


For GRIB2 data, simply use the script create_ncep_binary_stormcenter_grib2.py.

Step 6. Repeat Steps 4–5 for different forecast lead times, and ocean basins if needed. One can also repeat Steps 4–5 for different domain sizes to examine the sensitivity of TCG prediction to different input data domains, if desired. By default, the domain size is set to 18 × 18 degree.

Step 7. Verify the output files under the output directory dir_out. It should contain two sub-directories pos and neg under which there is an equal number of data files corresponding to TCG events during the period listed inside the NCEP/FNL data directory dir_ncep.

It should be mentioned that our current support for this binary dataset workflow is for the GRIB1/GRIB2 data format only. The scripts are however self-contained and they can be easily extended to other reanalyses datasets in the NETCDF format by using the same functions and packages, as the GRIB data package xarray is flexible and can handle both GRIB1/GRIB2 and NETCDF data format readily. Our code is designed and packaged, using the Python dependency management and packaging tool Poetry, which makes it easy to port to another platform for comparison and reproduction.

## Data Availability

Both the code and dataset presented herein are fully accessible on our GitHub repository at https://github.com/kieucq/tcg-binary-dataset. All Python codes follow the standard GNU Open Source Licence.
